# Dyke-Davidoff-Masson syndrome: case report of fetal unilateral ventriculomegaly and hypoplastic left middle cerebral artery

**DOI:** 10.1186/1824-7288-39-32

**Published:** 2013-05-14

**Authors:** Ettore Piro, Maria Piccione, Gianluca Marrone, Mario Giuffrè, Giovanni Corsello

**Affiliations:** 1 Department of Sciences for Health Promotion and Mother and Child Care “Giuseppe D’Alessandro”, University of Palermo, Palermo, Italy; 2 Department of Diagnostic and Interventional Radiology, Mediterranean Institute for Transplantation and Advanced Specialized Therapie, Palermo, Italy

**Keywords:** Dyke-Davidoff-Masson syndrome, Fetal ventriculomegaly, Cerebral hemiatrophy, Contrast enhanced-MRI angiography, Developmental delay, Hemiparesis

## Abstract

Prenatal ultrasonographic detection of unilateral cerebral ventriculomegaly arises suspicion of pathological condition related to cerebrospinal fluid flow obstruction or cerebral parenchimal pathology. Dyke-Davidoff-Masson syndrome is a rare condition characterized by cerebral hemiatrophy, calvarial thickening, skull and facial asymmetry, contralateral hemiparesis, cognitive impairment and seizures. Congenital and acquired types are recognized and have been described, mainly in late childhood, adolescence and adult ages. We describe a female infant with prenatal diagnosis of unilateral left ventriculomegaly in which early brain MRI and contrast enhanced-MRI angiography, showed cerebral left hemiatrophy associated with reduced caliber of the left middle cerebral artery revealing the characteristic findings of the Dyke-Davidoff-Masson syndrome. Prenatal imaging, cerebral vascular anomaly responsible for the cerebral hemiatrophy and the early clinical evolution have never been described before in such a young child and complete the acquired clinical descriptions in older children. Differential diagnosis, genetic investigations, neurophysiologic assessments, short term clinical and developmental follow up are described. Dyke-Davidoff-Masson syndrome must be ruled out in differential diagnosis of fetal unilateral ventriculomegaly. Early clinical assessment, differential diagnosis and cerebral imaging including cerebral MRI angiography allow the clinicians to diagnose also in early infancy this rare condition.

## Background

Dyke-Davidoff-Masson syndrome (DDMS) was first reported in 1933 [1]. Since then several patients have been described, mainly in late childhood, adolescence and adult ages. Main clinical features consist of cerebral hemiatrophy (CH), contralateral hemiplegia, facial asymmetry, developmental delay, cognitive impairment and seizures. Congenital and acquired presentations of DDMS are recognized. In the congenital type structural anomalies of the cerebral artery vessels have been recently described and considered responsible for the hemispheric atrophy. Few patients with DDMS have been diagnosed during infancy. To our knowledge our patient represents the first clinical description of a child with prenatal ventriculomegaly as indirect finding of cerebral parenchymal involvement in which Contrast-Enhanced MRI angiography allowed the earliest diagnosis of congenital DDMS. We describe the clinical course, brain imaging and neurophysiologic data collected from prenatal evidence of ventricular dilatation up to 9-months of age.

## Case report

**Figure 1:**
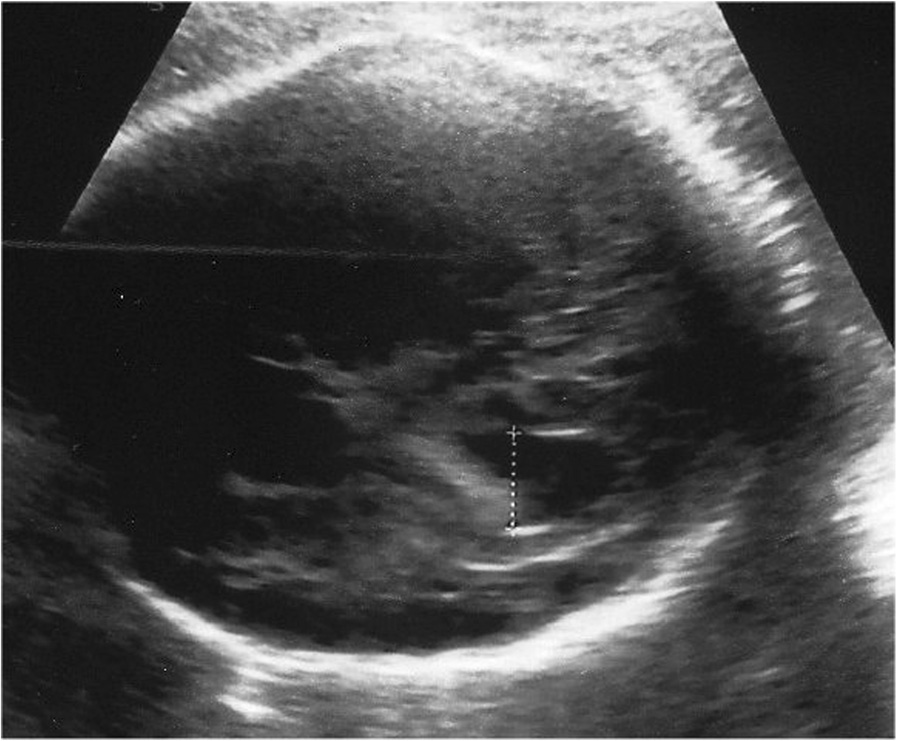
**Prenatal ultrasonographic image from our patient at 32 + 3 GW.** Left ventriculomegaly.

Our patient is a female second-born child of healthy non-consanguineous italian parents. The mother was short in stature (152 cm,< 3th centile) and reported no exposure to teratogens during pregnancy. No family history of birth defects and neurologic or cognitive impairment was reported. A fetal ultrasonography (US) at 32 + 2 weeks of gestation (GW) showed a left ventriculomegaly with atrial width of 11.8 mm (Figure 1). The child was born by spontaneous delivery at 38 + 4 GW. Her weight was 2670 g *(*10th centile*,* 50th centile for 36 weeks gestational age), her length was 43 cm (1th centile, 50th centile for 33 weeks gestational age) and the head circumference 31.2 cm (2th centile, 50th centile for 34 weeks gestational age). On admission she showed bilateral frontal bossing (present in the mother), a slight left occipital flattening, shortening of the neck, flat nasal bridge, rhizomelic shortening of four limbs and mild axial hypotonia with abdominal distention. The rest of the physical examination was unremarkable and no asymmetry of the body was present.

**Figure 2:**
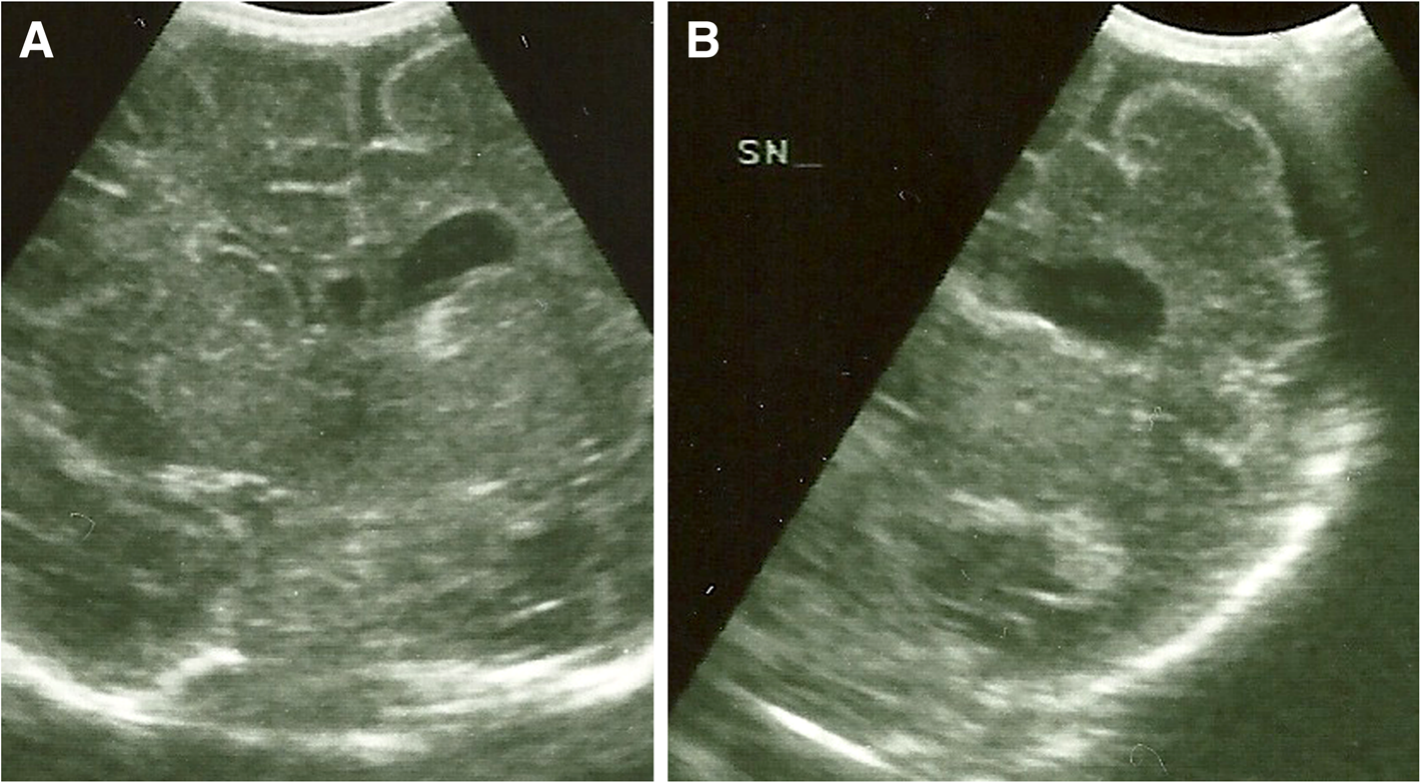
**Neonatal cerebral ultrasonographic coronal images. A** Left ventriculomegaly, **B** Left hemisphere atrophy with enlarged subarachnoid spaces.

Transcranial US showed a left hemisphere atrophy with enlarged subarachnoid spaces and ipsilateral ventriculomegaly (Figure 2A-B) Using Doppler ultrasound the left middle cerebral artery (MCA) was not visualized contrary to the right one. ToRCH complex infections were excluded, echocardiogram and abdominal US were normal. Mild axial hypotonia and weak suction persisted in the first week with normal spontaneous motility. Direct ophthalmoscopic examination of the fundus oculi was normal and light emitting diode goggle binocular and monocular flash visual evoked potentials (LED fVEP) showed a normal latency of the principal component (P200). At 21 days of life an electroencephalogram (EEG) revealed a normal background activity with a lower amplitude in the atrophic left hemisphere. A first brain MRI confirmed the hemispheric atrophy with left lateral ventricle dilatation, normal myelination and a thin corpus callosum. Owing to the reported dysmorphic features and to exclude the hypothesis of achondroplasia, karyotype, fibroblast growth factor receptor 3 (FGFR3) mutation analysis and array based genomic comparative hybridization (array CGH) were conducted, obtaining normal results. Blood ammonia and acylcarnitine were normal, urinary organic acids were absent. Conventional X-rays showed normal vertebral column and bones of hands and foot.

**Figure 3:**
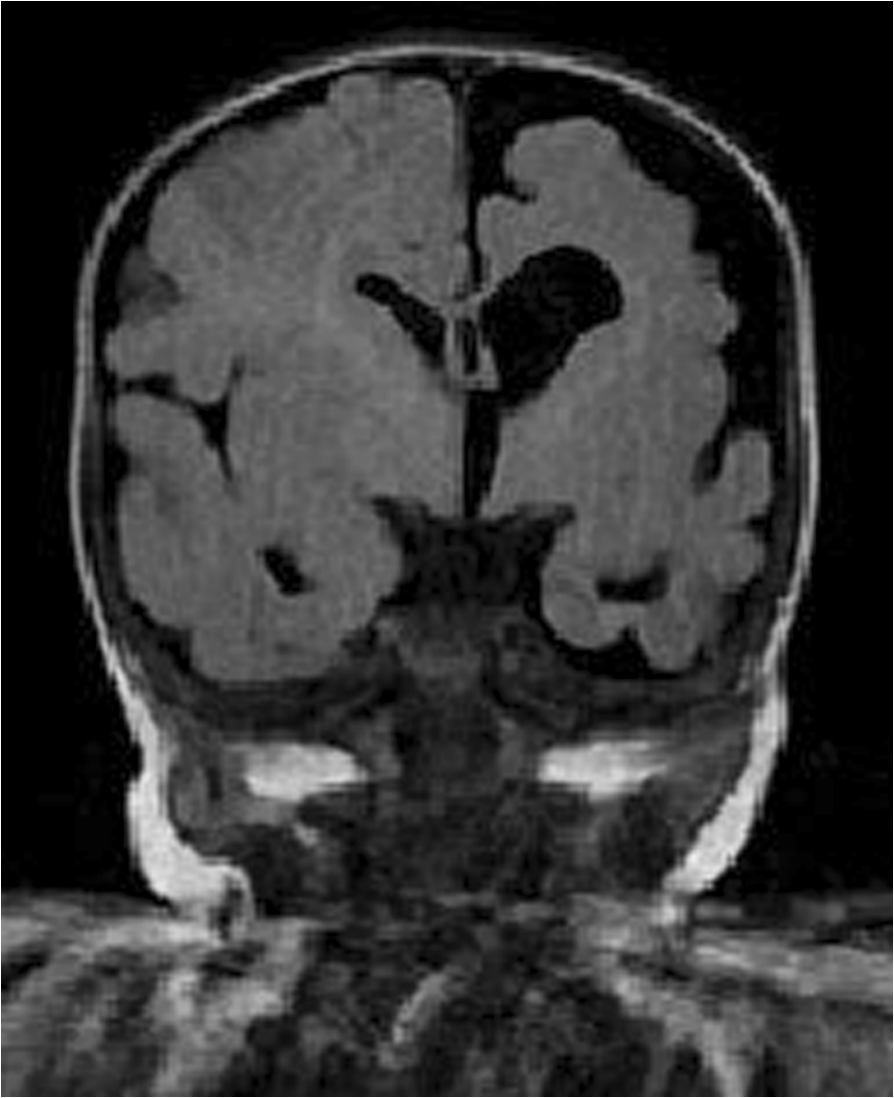
**Two-month-old patient.** Brain MRI coronal FLAIR image shows atrophy of the entire left hemisphere with compensatory ipsilateral midline structure shifting.

**Figure 4:**
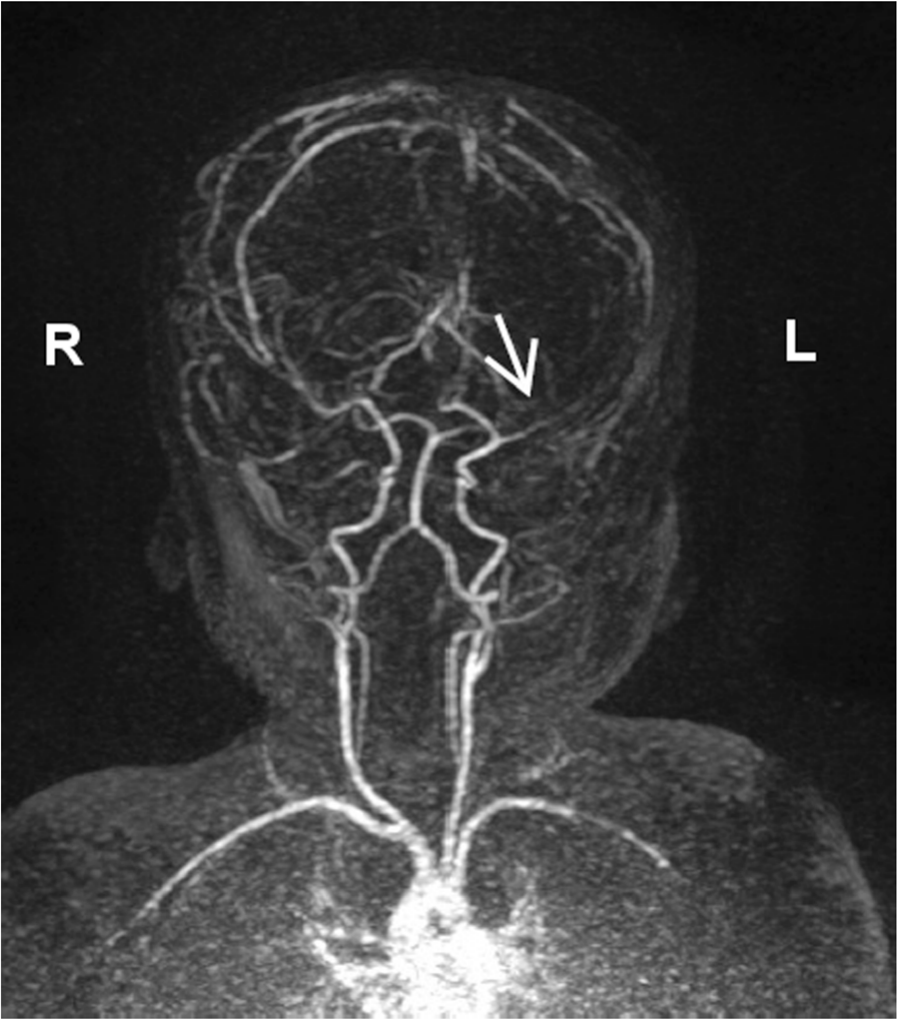
**Two-month-old patient.** Contrast enhanced-MRI-angiography. Three-dimensional multiplanar reconstruction image shows normal patency of the carotid arteries and hypoplasia of the left middle cerebral artery (white arrow).

At 2 months she showed a more evident isolated left occipital posterior flattening with no face asymmetry and mild axial hypotonia. Bilateral horizontal nystagmus was present, eye contact and social smiling were developed, but using a red woollen ball, eye fixation was delayed with incomplete ocular pursuit. LED fVEP showed a bilateral maturation. A second brain MRI revealed a left CH with homolateral enlargement of the frontal, occipital and temporal horns of the lateral ventricle with loss of grey matter, areas of polymicrogyria and to a lesser extent pachygyria in the left temporal-occipital region were suspected, ispilateral midline structure shifting to the left with left mild hypoplasia involving thalamus, caudate nucleus and lentiform nucleus, slight hypoplasia of the left cerebral peduncle and left calvarian thickening (Figure 3). No pathological enhancement was identified in the brain parenchyma. Brain contrast enhanced MRI angiography of the intracranial arteries showed normal development of the arteries of the neck and reduced caliber of the left MCA involving M1, M2 and M3 segments (Figure 4).

At 6 months of age a left facial asymmetry was emerging and the Bayley II Mental Developmental Index (MDI) was 65 (severely delayed). Nystagmus was no more present. Stretching toward objects, grasping and manipulation were present only with the left hand. She was able to sit with little back support, the sideways parachute reflex was absent on the right side. At 9 months of age her weight, length and head circumference were below the 3rd centile, left facial hypotrophy was evident and the Bayley II MDI was 58. Right upper and lower limbs hypotrophy was present. At rest the right arm was maintained in flexed position, early signs of contralateral hemiplegia. An EEG showed a normal background activity for age and a mild asymmetry in amplitude with a lower voltage in the left hemisphere.

## Discussion

Our report can be considered original since we describe the earliest clinical presentation and diagnosis of a primary or congenital type of DDM. Early diagnosis in this case could be achieved thanks to a group with experience in pediatric dysmorphology.

In our patient isolated unilateral ventriculomegaly, detected on prenatal US, was confirmed to be related to hemispheric hypotrophy on neonatal transcranial US and MRI. Transcranial Doppler US arose suspicion of left MCA anomaly so we performed a contrast enhanced-MRI angiography that confirmed the reduced caliber of the left MCA and its segments concomitant to unilateral brain hypoplasia.

Clinically apart from the isolated depression exhibited at birth, our patient did not suffer respiratory distress, encephalopathy or multiorgan dysfunction. She showed transient weak suction and an isolated axial hypotonia of central origin persistent at two months with a normal spontaneous motility. Thus until the second month of age, clinical evidence of hemisyndrome in terms of posture, asymmetry of resting muscular tone or spontaneous motility was not present. The normal latency of the principal fVEP component, in the presence of a left occipital lobe hypotrophy, is compatible with the supposed subcortical generators of fVEP [2]. Nystagmus, immaturity of eye fixation and pursuit may be ascribed to impairment of functionally related cortical and subcortical areas. The first peculiar findings of DDMS, face asymmetry and impaired left upper limb function, emerged by six months of age and at 9 months of age right sided hemiplegia and body hypotrophy affecting also the limbs were clearly evident. Body hypoplasia affecting principally the limbs, contralateral to the atrophic hemisphere and associated with superficial sensory deficit and hemiplegia, can be considered secondary to the impaired hemispheric functions.

This very early clinical evolution has never been described before and integrates with previous clinical reports of DDMS in children [3-5] that describe the neurodevelopmental impairment in patients aged at least 12 months.

In our patient isolated unilateral ventriculomegaly (atrial width 11,8 mm) was detected on prenatal US at 32 + 2 GW. Clinical series suggest that at between 15 and 40 GW, on prenatal ultrasound evaluation, an atrial width of < 10 mm is indicative of normal cerebral ventricular size. Ventriculomegaly corresponds to an atrial width of ≥ 10 mm and is considered mild when the atrial width is between 10 and 12 mm, moderate if 13-15 mm and severe when ≥ 16 mm [6]. Cerebral lateral ventricular asymmetry, with the two asymmetric atrial width of < 10 mm, is considered a normal variant and is prevalent in male [7]. Ventriculomegaly can be primarily related to blockage of cerebrospinal fluid outflow through the foramen of Monro or ipsilateral reduced brain tissue with concomitant ex-vacuo ventricular enlargement. Shen et al. described three patterns of CH on MRI: pattern I, diffuse cortical and subcortical atrophy; pattern II, diffuse cortical atrophy associated with expanded porencephalic cysts; and pattern III, old infarction with necrosis in the territory of the MCA [8]. In our patient at two months of age brain MRI showed a pattern I of CH with peculiar bone and parenchymal changes. Concomitant encephalomalacia has been detected in two of the four reported one-year-old patients with DDMS [4]. In our patient no hypoxic ischemic parenchymal lesion was present. Several pathological conditions are associated with fetal/neonatal CH (Table 1). Cerebral hemispheric hypoperfusion can be due to aortic coarctation, internal carotid hypoplasia or agenesia, and reduced or absent middle cerebral artery (MCA) and/or its ramifications development [9]. On the basis of the described congenital vascular changes, hemispheric hypoperfusion, occurring during early brain development, is considered in literature the prevalent etiopathogenic hypothesis for CH in the congenital type of DDMS. DDMS and cerebral migration disorders are associated with abnormalities that affect the development of cerebral arteries and the persistence of embryonic arteries, Dandy Walker malformation and cerebellar malformations, as well as congenital vascular abnormalities, such as facial hemangioma and reticulated capillary malformations have been described [10]. These findings suggest a common angiogenetic developmental anomaly occurring during the same embryonic period, approximately 4-5 weeks [11]. Male sex and left hemisphere predisposition to vascular impairment has been reported in DDMS [3]. Ipsilateral polymicrogyria, mesencephalon and brainstem hypoplasia and crossed cerebellar atrophy are occasionally present [12-14]. At two months of age a contrast enhanced-MR angiography showed a normal appearing of the arteries of the neck (internal carotid arteries and vertebral arteries) and intracranially a reduced caliber of the left middle artery cerebral involving the M1, M2 and M3 segments. The posterior cerebral arteries were within normal limits. Congenital middle cerebral artery hypoplasia must be differentiated from cerebral artery occlusion predisposing to arterial stroke due to thromboembolic events, infectious and non–infectious inflammatory conditions, genetic pathologies, inborn errors of metabolism. Diagnosis of fetal, neonatal, or presumed fetal or neonatal stroke can be made on the basis on clinical history, physical examination, laboratory findings and brain imaging showing peculiar localization and evolution of parenchymal infarct [15].

**Table 1:** Congenital and acquired conditions associated with fetal/neonatal cerebral hemiatrophy

**Congenital**	**Acquired**
Cerebral hemispheric hypoperfusion [11]	Periventricular leucolamalacia,
Brain dysgenesis (schizencephaly, polymicrogyria)	Cerebral hemorrage
Septo-optic dysplasia	Cerebral infarction
Sturge-Weber syndrome	Cerebral infection
Silver-Russel syndrome	
Encephalocraniocutaneous lipomatosis [9]	
Schimmelpenning syndrome [10]	

## Conclusions

Dyke-Davidoff-Masson syndrome must be ruled out in differential diagnosis of fetal unilateral ventriculomegaly. We report the earliest diagnosis of Dyke-Davidoff-Masson syndrome described in infancy. Early clinical assessment, differential diagnosis and cerebral imaging including cerebral MRI angiography allow the clinicians to diagnose also in early infancy this rare condition.

## Consent

Written informed consent was obtained from the patient's parents for publication of this report and any accompanying images.

## Competing interests

The authors declare that they have no competing interests.

## Authors’ contributions

EP performed clinical and neurological assessment, formulated the diagnosis, was involved in drafting the manuscript and revising it,.MP performed genetic counseling and was involved in drafting the manuscript, MG performed genetic counseling and was involved in drafting the manuscript, GM performed cerebral MRI and angiography and was involved in drafting the manuscript, GC performed clinical assessment, revising it critically and contributed to the final version of the paper. All authors read and approved the final manuscript.
